# Development and Evaluation of a Mindfulness-Based Mobile Intervention for Perinatal Mental Health: Randomized Controlled Trial

**DOI:** 10.2196/56601

**Published:** 2025-01-17

**Authors:** Sehwan Park, Hee Young Cho, Jin Young Park, Kyungmi Chung, Kyungun Jhung

**Affiliations:** 1 Biomedical Research Institute Catholic Kwandong University International St. Mary’s Hospital Incheon Republic of Korea; 2 Medical Research Team Digital Medic co., Ltd. Seoul Republic of Korea; 3 Department of Obstetrics and Gynecology Seoul National University College of Medicine Seoul Republic of Korea; 4 Department of Psychiatry, Yongin Severance Hospital Yonsei University College of Medicine Yonsei University Health System Yongin Republic of Korea; 5 Institute of Behavioral Science in Medicine Yonsei University College of Medicine Yonsei University Health System Seoul Republic of Korea; 6 Center for Digital Health, Yongin Severance Hospital Yonsei University College of Medicine Yonsei University Health System Yongin Republic of Korea; 7 Department of Psychiatry Catholic Kwandong University International St. Mary’s Hospital Catholic Kwandong University College of Medicine Incheon Republic of Korea

**Keywords:** anxiety, perinatal mental health, depression, mobile health care, mindfulness, mobile phone

## Abstract

**Background:**

Perinatal mental health problems, such as anxiety, stress, and depression, warrant particularly close monitoring and intervention, but they are often unaddressed in both obstetric and psychiatric clinics, with limited accessibility and treatment resources. Mobile health interventions may provide an effective and more accessible solution for addressing perinatal mental health. Development and evaluation of a mobile mental health intervention specifically for pregnant women are warranted.

**Objective:**

This study aimed to evaluate the effectiveness of a 4-week, self-administered mobile mindfulness intervention in reducing anxiety, depression, and stress, and improving emotional well-being, maternal-fetal attachment, and mindfulness skills in a general population of pregnant women.

**Methods:**

Pregnant women were recruited and randomized to an intervention or a wait-list control group. The intervention group participated in a self-administered 4-week smartphone-based mindfulness program. Anxiety, depression, and stress were assessed as primary outcomes at baseline and postintervention. Secondary outcomes were mental health well-being, maternal-fetal attachment, and skills of mindfulness. The usability of the mobile intervention was also evaluated.

**Results:**

A total of 133 pregnant women were randomly assigned to the intervention (n=66) or the control group (n=67). The overall dropout rate was 30% (39/133). Anxiety scores of the intervention group significantly decreased from baseline to postintervention (*P*=.03, Wilcoxon Signed-Rank test), whereas no significant changes were observed in the control group. Depression and stress scores showed no significant changes. Emotional well-being significantly improved in the intervention group (*P*=.01). Improvements were observed in maternal-fetal attachment, particularly in attributing characteristics to the fetus (*P*=.003) and in differentiating the self from the fetus (*P*=.006). Mindfulness awareness also showed significant improvement (*P*=.008). Significant between-group effects were identified for mindfulness awareness (*P*=.006) and attributing characteristics to the fetus (*P*=.002). After applying the false discovery rate corrections, within-group improvements in emotional well-being, maternal-fetal attachment, and mindfulness awareness remained significant, while between-group differences for emotional well-being and differentiation were not significant.

**Conclusions:**

A mobile mindfulness program effectively reduced anxiety and improved emotional well-being, maternal-fetal attachment, and mindfulness awareness in the general population of pregnant women. Mobile interventions may offer a cost-effective and feasible method for promoting perinatal mental health.

**Trial Registration:**

Clinical Research Information Service KCT0007166; https://tinyurl.com/458vfc4r

## Introduction

Pregnancy is a major period of change in a woman’s life. Perinatal women are reported to be at a higher risk of developing newly-onset mental health problems or experiencing a relapse of existing ones compared with the general population [1]. The percentage of women in pregnancy who meet 1 or more diagnostic criteria for an anxiety disorder ranges from 12.2% to 39%, which is 2-3 times higher than the general population [2]. Furthermore, the prevalence of antenatal depression is estimated at 20.7% for any depression and 15% for major depression [3]. Stress associated with pregnancy and childbirth is known to increase the risk of perinatal anxiety and depression, and the risk is increased in those with previous mental health vulnerabilities [4]. Concerning such widespread prevalence, the World Health Organization (2024) emphasizes the need for health care professionals to recognize and provide adequate interventions for mental health problems in pregnant women [5].

Mental health problems during pregnancy have far-reaching effects, influencing both the immediate and long-term outcomes of mothers and their children [6,7]. Antenatal depression and anxiety increase the risk of pregnancy complications and obstetric outcomes such as preeclampsia, edema, hemorrhage, preterm birth, and low birth weight [8]. Negative mental health conditions during pregnancy can hinder self-care, increasing risks such as poor nutrition and substance misuse. Consequently, these factors may harm the physical and mental health of the mother and restrict healthy fetal growth and development [9,10]. Antenatal depression also increases the risk of postnatal depression [11], which will further impact the health of both the mother and the infant. In the long term, untreated perinatal mental health conditions can lead to lasting consequences such as impaired neurocognitive development and poor emotional regulation during infancy and childhood [12-14].

Despite the significant impact of perinatal mental health issues, many women face barriers that prevent them from accessing necessary mental health care [15]. In obstetric clinics, routine mental health evaluations for anxiety, depression, and stress are often neglected or overlooked, and it is uncommon for pregnant women to seek mental health care directly from psychiatric services. Stigma further discourages help-seeking in pregnant women, and finally, there is a lack of available resources. Even when clinical problems are identified, many pregnant women are reluctant toward pharmacotherapy due to concerns about fetal safety and the potential effects of medication [16,17]. This low acceptability of pharmacotherapy emphasizes the need for nonpharmacological approaches, which many pregnant women may find more acceptable. However, access to nonpharmacological treatments, such as psychotherapy, remains limited due to resource constraints, including availability and cost. Given these barriers, there is a clear need for effective and more accessible nonpharmacological interventions for women during this critical period, including digital and mobile-based programs, that can overcome obstacles like stigma and limited resources.

The integration of digital technology in psychotherapeutic interventions for perinatal women has shown promising results. A systematic review and meta-analysis by Lau et al [18] showed that digital psychotherapeutic interventions may significantly reduce symptoms of depression, anxiety, and stress in perinatal women. This review emphasized that interventions delivered through digital platforms, such as websites and mobile apps, were particularly effective in improving psychological outcomes [18]. A mobile or web-based approach may alleviate space-time barriers, overcome women’s resistance to treatment, and address limited resources. Research indicates that perinatal women frequently use mobile apps to access information about pregnancy, childbirth, and childcare [19]. These results, along with others, show that many women find mobile apps highly accessible and usable for improving their self-care and enhancing their pregnancy experience [20]. Mobile health apps have been reported to significantly enhance areas of both treatment and health care delivery [21]. Therefore, employing mobile and web-based technologies provides a promising way to address the unique needs of perinatal women, improving their access to adequate care.

Mindfulness-based interventions (MBIs) have emerged as an effective method for psychological self-care and symptom management [22]. By fostering conscious awareness and acceptance of one’s present state, mindfulness equips individuals with the ability to navigate through physical and emotional distress [23]. Numerous studies highlight its efficacy in reducing anxiety and depression symptoms, as well as enhancing overall psychological well-being [24-28]. Mindfulness-based stress reduction and mindfulness-based cognitive therapy (MBCT) have shown efficacy in reducing anxiety and preventing depression relapse, as well as enhancing a broad range of other mental health problems [29,30].

Mindfulness training techniques through mindfulness-based stress reduction or MBCT have been shown to be effective for pregnant women in reducing stress, anxiety, depressive symptoms, and emotional distress during pregnancy [26,28,30,31]. A systematic review on the effectiveness of MBIs on maternal perinatal mental health outcomes showed reductions in perinatal anxiety and less consistent results for the effect on depression, but the review was limited by the number of studies available and their methodological quality [25]. Even though mindfulness interventions may be effective in perinatal mental health care, gold-standard mindfulness training often requires rigorous hours of in-person instruction and training. Thus, traditional mindfulness programs pose similar accessibility barriers to pregnant women.

Mobile-based MBIs may offer a promising solution to overcoming these barriers in perinatal mental health care [32-35]. Sun et al [33] implemented an 8-week mindfulness program, which was shown to be effective for pregnant women who screened positive for depressive symptoms. In the study, only 8% (7/84) completed the entire 8-week training program. As smartphone-based mobile interactions are mostly self-administered, the acceptability of the intervention by the participant is an important part of treatment viability. We aimed to address this point by developing a shorter, more usable self-administered mobile MBI that is effective for perinatal mental health. Furthermore, increased depression, anxiety, and stress during pregnancy are associated with negative prenatal outcomes, even at a subclinical level. Subclinical anxiety and stress were linked to increased risks of preterm birth, low birth weight, and lower Apgar scores [36]. In addition, mild depressive symptoms are reported to be associated with heightened cortisol levels, impacting fetal neurodevelopment and increasing the likelihood of neonatal complications [36]. This suggests that addressing even mild symptoms in pregnancy could improve maternal and neonatal health outcomes. Thus, in developing the intervention, we aimed to target the general population of pregnant women to improve their mental health and well-being, compared with previous studies that often focus on the clinical population.

In this randomized controlled trial, we evaluated the effectiveness of our newly developed, 4-week mindfulness-based mobile app in reducing perinatal anxiety, depression, and stress among a general population of pregnant women. We also investigated mental health well-being, maternal-fetal attachment, and mindfulness skills as secondary outcomes, and assessed the usability of the intervention to determine feasibility for broader application.

## Methods

### Trial Design and Randomization

This study was a single-center, randomized wait-list controlled trial with assessments at baseline and post intervention (trial registration KCT0007166). Participants were randomized using a list created in the R (4.0.2 version; R Core Team) program, which was set to randomize in a 1:1 ratio. To ensure the integrity of the data analysis and eliminate potential bias, the data analysts remained blinded to the group allocation of each participant. The randomization was performed by a designated member of the research team who was not involved in the data analysis. The randomization sheet was only accessible to the person responsible for assigning participants to their respective groups. A designated member of the research team allocated the participants to the intervention or control group using the randomized list. Those randomly assigned to the wait-list control group received the intervention after 4 weeks. The reporting of this trial adheres to the CONSORT-EHEALTH (Consolidated Standards of Reporting Trials of Electronic and Mobile Health Applications and Online Telehealth) guidelines (Multimedia Appendix 1).

### Sample Size

The sample size was determined using G*Power (version 3.1.9.7), based on standard assumptions for detecting differences between 2 groups over time. The calculation assumed a small-to-medium effect size and a statistical power of 80%, with the required sample size estimated at 90 participants (45 per group) [18,25]. To account for potential attrition, which has been reported to reach up to 33% in similar interventions [18,25], the recruitment target was set at 134 participants.

### Participants

Participants were recruited both from internet-based advertisement and on-site at the Obstetrics and Gynecology Department of the CHA Bundang Medical Center in Seongnam, South Korea, from March to November 2020. The hospital is the largest and first specialized center for pregnancy and women’s health in the country. Informational posters displayed in the hospital and internet-based advertisements were used for recruitment. Potential participants were provided with detailed information about the study, and those interested were invited to contact the research team for further screening and enrollment. The inclusion criteria were as follows: (1) at least 18 years of age, (2) between 1 and 32 weeks of gestation, (3) having a smartphone, (4) being able to use the app on a smartphone for the study, (5) being able to read and understand Korean, (6) willing to be randomized, and (7) willing to provide informed consent. Exclusion criteria were as follows: (1) over 32 weeks of gestation and (2) expected to give birth within 4 weeks (during the intervention period). The study did not exclude participants based on medical history (eg, obstetric complications or mental illness) unless these conditions reached a clinically significant level that would affect participation or safety of the participant.

Upon obtaining informed consent and ensuring random allocation, all participants completed a baseline evaluation, including sociodemographic, medical, pregnancy-related, and mental health information. If participants could not visit the obstetric department or complete the questionnaire at the site, they received a Google survey link of the questionnaire through email or smartphone.

Participants assigned to the intervention group were instructed to download a mobile-based mindfulness intervention app named AvecMom (Digital Medic Co) and were instructed to use it for 4 weeks. They received printed guides on how to use the mobile app and direct download links for both Android (Google) and iPhone (Apple Inc) devices. Participants were instructed to practice each practice session at least 3 times a week for the 4 weeks of participating in the program.

Participants assigned to the wait-list control group were instructed not to participate in any events of meditation, yoga, or mindfulness-based activities during the 4 weeks. After the 4-week period, all participants received a Google survey link for a postintervention assessment through email, messenger, or in person at the site. The control group received access to the mobile app with instructions after the 4 weeks and the postintervention assessment.

### Ethical Considerations

This study was approved by the institutional review board, CHA Bundang Medical Center, CHA University College of Medicine (HI18C0911). All participants provided written informed consent before enrollment. Participants were fully informed about the study’s purpose, procedures, potential risk, and their right to withdraw at any time without consequence. To ensure privacy and confidentiality, all data were deidentified before analysis and securely stored on encrypted servers, accessible only to authorized research staff. For data with potential reidentification risks, access was strictly limited to authorized research staff. Comprehensive data management protocols were implemented to prevent unauthorized access and ensure participant confidentiality throughout the study. Participants received pregnancy-related gifts, valued at approximately US $41 (50,000 KRW), as an incentive for their participation in the study.

### Development of the Mobile-Based Mindfulness Intervention

The mindfulness-based mobile intervention was developed by an interdisciplinary research team. The development was led by a professor of psychiatry who has over 5 years of mindfulness experience as well as experience with leading the development of several preventive interventions for mental health-related at-risk populations. The team also consisted of a psychiatrist with expertise in mobile apps, obstetricians, psychologists, a research professor of user interface and user experience design, and research assistants with mindfulness experience. The development process of the intervention began with a comprehensive review of previous MBIs to identify essential components relevant to perinatal mental health [37]. Following this, we focused on component selection, choosing mindfulness practices that address perinatal stress, anxiety, and depression, with content tailored to meet the unique physical, social, and psychological needs of pregnant women. To ensure feasibility and accessibility, the program was designed as a 4-week intervention, a modification supported by existing research on shorter programs’ effectiveness [38]. Finally, the program underwent expert review by a panel of experienced advisors in mindfulness and perinatal mental health, whose insights helped refine the content and structure. The recorded sessions were guided by an experienced mindfulness trainer who was also pregnant at the time, strengthening the intervention’s relevance and the empathetic element of the intervention. Participants were reminded that this app is not equivalent to psychotherapy, and they were recommended to seek professional help when necessary.

The intervention program consisted of 4 sections—breathing mindfulness meditation, body scanning, emotional awareness, and self-kindness mindfulness (Multimedia Appendix 2). Each session was divided into 2 subsessions—instruction and practice sessions. In the instruction session, participants learned about mindfulness techniques according to the thematic curriculum and were guided through the mindfulness training. In the practice session, participants entered the mindfulness practice directly without the introduction content of the instruction session. Participants needed to complete the instruction session to enter the practice session. The sessions were designed to be completed sequentially, with the subsequent session automatically unlocking once the participant performs the previous session at least twice. This sequence was clearly outlined in the user manual provided to the participants.

Each session had a duration of about 20 minutes. Participants were instructed to practice each session at least twice, ensuring they engaged with the content multiple times to reinforce their learning and practice. This structured approach aimed to enhance usability and effectiveness, providing a comprehensive yet manageable intervention for pregnant women. Sessions took into consideration the prenatal-specific psychological, physical, and emotional changes specific to pregnancy. After each session, participants received a sequential worksheet to complement the session and aid in understanding and developing mindfulness skills (Multimedia Appendix 3).

### Measures

#### Depression, Anxiety and Stress Scale–21 Items

Depression, anxiety, and stress were assessed with the 21-item Depression, Anxiety and Stress Scale (DASS-21) [39-41]. This scale consists of 21 items used to assess depression (7 items), anxiety (7 items), and stress (7 items). The response items were scored on a 4-point Likert scale ranging from 0 to 3. Higher scores for each factor indicate a higher intensity for each symptom. A depression score of 9 or higher, an anxiety score of 7 or higher, and a stress score of 14 or higher indicate moderate symptoms for each factor [40]. The internal reliability for DASS-21 was found to be high (α=.94, at baseline).

#### Edinburgh Postnatal Depression Scale

The Edinburgh Postnatal Depression Scale (EPDS) [42,43] was also used to assess depressive symptoms during the peripartum period. This scale contains 10 items scored using a 4-point Likert scale ranging from 0 to 3. A higher score indicates a higher intensity of depressive symptoms. A score of 8 or less on the recommended cutoff indicates no likelihood of depression [42]. The reliability of the EPDS for pregnant Korean women was good (Cronbach α=0.85) [43]. The scale reached good-to-excellent internal reliability in our sample (at baseline, Cronbach α=0.88). Although the EPDS is a frequently used measure to assess postnatal depression, it has been used in many studies as a valid screening tool to detect depression in pregnant women [44,45].

#### Mental Health Continuum-Short Form

Mental health well-being was measured using the Mental Health Continuum-Short Form (MHC-sf) [46,47]. This scale consists of 14 items measuring social (5 items), psychological (6 items), and emotional (3 items) well-being, which is scored using a 6-point Likert scale ranging from 0 to 5. Higher scores indicate better mental health. The 3 MHC-sf subscales had high test-retest reliability, discriminant validity, and convergent validity in Korean adults [47]. Adequate reliability was found in this study (Cronbach ⍺=0.096, at baseline).

#### Maternal-Fetal Attachment Scale

Attachment between the mother and fetus was assessed using the Maternal-Fetal Attachment Scale (MFAS) [48]. In this study, 23 items of the revised Korean version were used, which were scored using a 4-point Likert scale. The 5 subscales of the MFAS are designed to measure differentiation of self from the fetus (3 items), interaction with the fetus (4 items), attributing characteristics to the fetus (6 items), giving of self (4 items), and maternal role-taking (6 items). Construct and criterion validity and reliability have been reported for pregnant Korean women [49]. The study’s α coefficient for the baseline MFAS score was 0.92.

#### Cognitive and Affective Mindfulness Scale-Revised Version

Mindfulness was assessed with the Cognitive and Affective Mindfulness Scale-revised (CAMS-r) [50,51]. The scale contains a 10-item inventory. The response items were scored on a 4-point Likert scale ranging from 1 to 4. The CAMS-r consists of 3 factors that assess mindfulness levels—awareness, attention, and acceptance. The awareness and attention factors comprise 4 items, and acceptance consists of 2 items. Higher scores indicated higher levels of mindfulness. The CAMS-r has good internal reliability (Cronbach α=0.73), internal validity (Cronbach α=0.70), and test-retest reliability (*r*=0.77, *P*<.001) among Korean undergraduate students [51]. This study’s α coefficient for the baseline CAMS-r score was 0.86.

#### Usability

To assess the usability of the mindfulness mobile app, the Usefulness, Satisfaction, and Ease of Use questionnaire (USE questionnaire) [52] was used after 4 weeks of using the app. This scale consists of 30 items measuring usefulness (8 items), ease of use (4 items), ease of learning (11 items), and satisfaction (7 items). The scale used was a 7-point Likert scale ranging from strongly disagree (1) to strongly agree (7). The reliability of the questionnaire in this study was assessed using Cronbach α, with results demonstrating high internal consistency across all 4 dimensions (usefulness: 0.914, ease of use: 0.898, ease of learning: 0.883, satisfaction: 0.905). These findings are consistent with those reported by Gao et al [53], who found similarly high reliability (Cronbach α=0.98 overall).

### Data Analysis

All statistical analyses were conducted using SPSS (version 18.0; IBM Corp) and Python 3 (Python Software Foundation). Demographic characteristics were analyzed in SPSS 18.0. An independent samples *t* test was used for continuous variables, while categorical variables were analyzed using the chi-square test to ensure baseline comparability between the intervention and control groups.

For demographic variables, missing data points related to smoking (1 participant) and alcohol use (2 participants) were imputed using the last observation carried forward method [54]. This method involved using the most recent patient records to impute the missing values, ensuring completeness of the demographic dataset for analysis.

The primary analysis was conducted using an intent-to-treat approach, ensuring that all randomized participants were included in the analysis based on their originally assigned groups. Due to the nonnormal distribution of the primary and secondary outcome data, nonparametric methods were applied using Python 3. The Mann-Whitney *U* test was used to compare outcomes between the Intervention and Control groups, while within-group changes from pre- to postintervention were assessed using the Wilcoxon Signed-Rank test.

Effect sizes for both the Mann-Whitney *U* and Wilcoxon tests were calculated using the following formula:



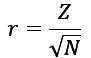



where Z is the test statistic and N is the number of observations. Effect sizes were interpreted based on the following guidelines: small (*r*=0.1), medium (*r*=0.3), and large (*r*=0.5).

To account for the nonparametric nature of the data, 95% CIs for the effect sizes were calculated using the bootstrapping method with 1000 resamples. These CIs provide a measure of precision, indicating the range within which the true effect size is likely to lie with 95% CI.

For secondary outcomes, multiple comparisons were addressed using the false discovery rate (FDR) correction method to control for Type I errors [55]. This method ensures that the statistical significance of the results is maintained while minimizing the risk of false positives due to multiple testing, particularly in the analysis of secondary outcomes.

All statistical results, including test statistics, *P* values, effect sizes, and 95% CIs, are reported to provide a comprehensive understanding of the intervention’s effectiveness.

## Results

### Participant Enrollment and Flow

**Figure 1:**
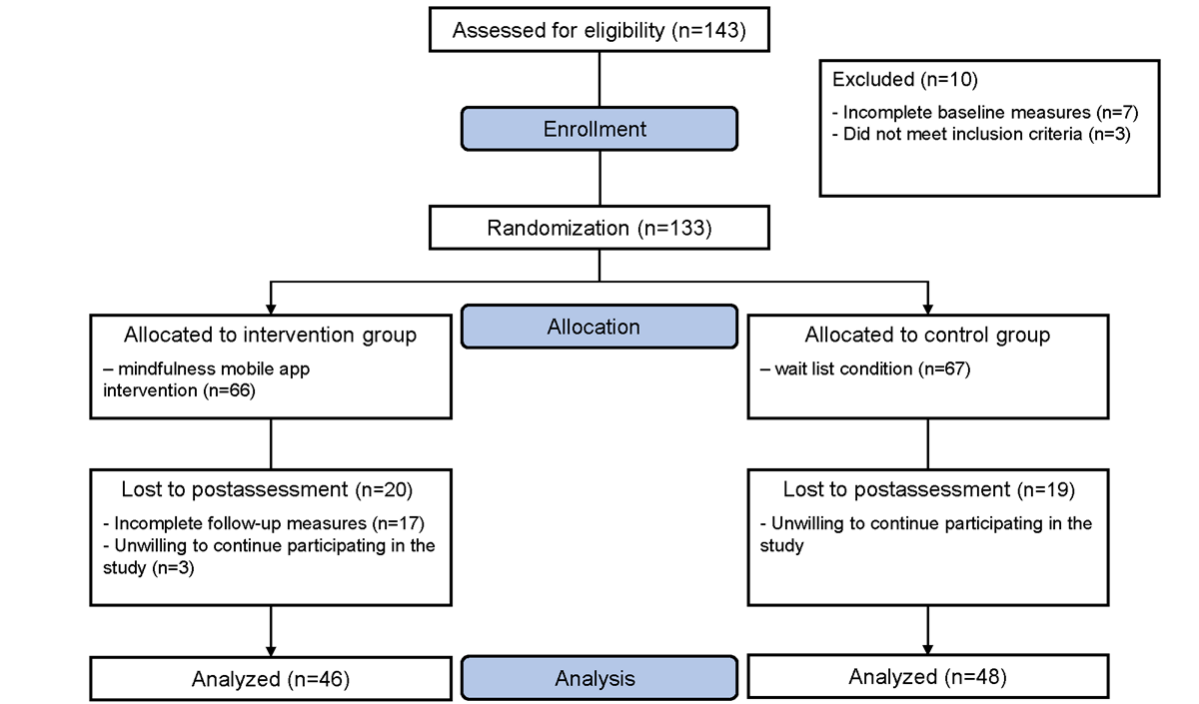
Participant enrollment flowchart.

A total of 143 perinatal women were recruited as eligible for the study. Of the 143 participants, 2% (3/143) did not meet the inclusion criteria, and 5% (7/143) did not complete the baseline assessment. Thus, 133 participants were allocated randomly, of which 66 were allocated to the intervention group and 67 to the wait-list control group. During the follow-ups, 13% (17/133) did not complete the follow-up assessment and 17% (22/133) were not willing to continue participation. The overall completion rate was 71% (94/133; Figure 1; see Multimedia Appendix 1 for the CONSORT-EHEALTH checklist).

### Demographics and Clinical Characteristics

Demographic characteristics are presented in Table 1. All 133 participants were pregnant and married at baseline. The mean ages of the participants for the intervention (n=66) and control groups (n=67) included in the analysis were 32.6 (SD 4.3) years and 32.8 (SD 4.44) years, respectively, and the mean gestational weeks of the 2 groups were 20.61 (SD 6.42) months and 20.04 (SD 6.76) months, respectively. There were no significant differences between the 2 groups across all the baseline characteristics, including pregnancy period, occupation, education level, income, pregnancy plan, medical diagnosis, and other characteristics.

**Table 1:** Demographics and clinical characteristics of study participants (n=133).

Characteristics		Intervention (n=66)	Control (n=67)	Test value (*df*)	*P* value
Age (years), mean (SD)		32.6 (4.3)	32.8 (4.44)	0.251^a^ (92)	.80
**Pregnancy period, n** **(%)**				2.3^b^ (2)	.32
	First trimester (0-13 weeks)	15 (23)	11 (16)		
	Second trimester (14-28 weeks)	43 (65)	42 (63)		
	Third trimester (29-42 weeks)	8 (12)	14 (21)		
**Occupation, n (%)**				7.9^b^ (7)	.34
	Housewife	35 (53)	32 (48)		
	White-collar worker	16 (24)	22 (33)		
	Services	3 (4)	4 (6)		
	Professional	7 (11)	5 (7)		
	Technical	2 (3)	0 (0)		
	Student	1 (2)	0 (0)		
	Self-employed	2 (3)	1 (1)		
	Other	0 (0)	3 (5)		
**Education, n (%)**				8.3^b^ (3)	.08
	Elementary School	0 (0)	1 (2)		
	Middle School	4 (6)	0 (0)		
	High school	13 (20)	7 (10)		
	University	46 (70)	53 (79)		
	Postgraduate degree	3 (4)	6 (9)		
**Monthly Income (in US $)^c^, n (%)**				1.96^b^ (4)	.74
	≤820	0 (0)	1 (1)		
	821-1639	1 (2)	2 (3)		
	1640-2459	16 (24)	14 (21)		
	2460-3279	21 (32)	18 (27)		
	>3280	28 (42)	32 (48)		
**Marriage status, n** **(%)**				—^d^	—
	Divorced, single, or separated	0 (0)	0 (0)		
	Married	66 (100)	67 (100)		
**Planned pregnant, n** **(%)**				1.5^b^ (1)	.23
	No	44 (67)	51 (76)		
	Yes	22 (33)	16 (24)		
**Smoking, n** **(%)**				0^b^ (1)	.99
	No	64 (97)	65 (97)		
	Yes	2 (3)	2 (3)		
**Alcohol use, n** **(%)**				1.1^b^ (1)	.29
	No	60 (91)	64 (96)		
	Yes	6 (9)	3 (4)		
**Depression history, n** **(%)**				.3^b^ (1)	.57
	No	57 (86)	60 (90)		
	Yes	9 (14)	7 (10)		
**Medical problems, n** **(%)**				2.9^b^ (1)	.09
	No	52 (79)	60 (90)		
	Yes	14 (21)	7 (10)		

^a^Independent *t* test.

^b^Chi-square test.

^c^All monetary values are presented in US $. The conversion rate used is US $1=1220.14 KRW, reflecting the exchange rate at the time of the study.

^d^Not applicable.

### Effectiveness of the Mobile-Based Mindfulness Intervention

#### Primary Outcomes: Anxiety, Depression, and Stress

Table 2 shows the mean scores of pre- and postmeasures of the DASS-21, EPDS, CAMS-r, MHC-sf, and MFAS in the intervention and control groups. The DASS-21 mean scores of the intervention group at preintervention were 6.7 (SD 7.2), 6.9 (SD 5.5), and 9.7 (SD 8.2) for depression, anxiety, and stress, respectively. The mean scores of depression, anxiety, and stress of the compared control group were 7.4 (SD 7.7), 6.9 (SD 7.3), and 11.7 (SD 8.6), respectively. Considering the recommended cutoff scores from previous research, mean scores for depression, anxiety, and stress were in the normal range [42]. The baseline mean scores of the EPDS in the intervention and control groups were 7.1 (SD 5.1) and 7.6 (SD 6.3), respectively.

**Table 2:** Comparison of depression, anxiety, and stress scores at pre- and postintervention time points between the intervention and control groups using nonparametric analysis.

Scale, variable, and group				Pretest				Posttest				*P* value^a^		*P* value^b^
				Mean (SD)		Median (IQR)		Mean (SD)		Median (IQR)				
**DASS-21^c^**														
	**Anxiety**													
		IG^d^	6.9 (5.5)		6 (2-10)		5.6 (5.8)		4 (2-7.5)		.03		.08	
		CG^e^	6.9 (7.3)		5 (2-8)		8.3 (8.2)		8 (2-10.5)		.11		—^f^	
	**Depression**													
		IG	6.7 (7.2)		4 (2-9.5)		6.2 (6.3)		4 (2-8)		.60		.13	
		CG	7.1 (7.6)		4 (2-10.5)		8.5 (7.8)		7 (2-12)		.09		—	
	**Stress**													
		IG	9.7 (8.2)		8 (4-13.5)		9 (8)		8 (2-13.5)		.48		.15	
		CG	11.7 (8.6)		10 (4-16)		11.6 (9.1)		10 (4-18)		.98		—	
**EPDS^g^**														
		IG	7.1 (5.1)		6 (4-9)		6.2 (4.6)		6 (2-9.75)		.16		.11	
		CG	7.6 (6.3)		6 (2-10.5)		8.3 (6)		8 (3.75-11.25)		.29		—	

^a^*P* value for Wilcoxon Signed-Rank test for within-group comparisons (pre- to posttest).

^b^*P* value for Mann-Whitney *U* test for between-group comparisons (posttest).

^c^DASS-21: Depression, Anxiety and Stress Scale, 21-question version.

^d^IG: intervention group.

^e^CG: control group.

^f^Not applicable.

^g^EPDS: Edinburgh Postnatal Depression Scale.

Anxiety scores, measured using the DASS-21, significantly decreased in the intervention group from pre- to postintervention, with the median score dropping from 6 (IQR 2-10) to 4 (IQR 2-7.5), as shown by the Wilcoxon Signed-Rank test (*W*=295, *P*=.03, *r*=6.413, 95% CI –2 to 5). In contrast, no significant changes were observed in the control group, where anxiety scores increased from a median of 5 (IQR 2-8) to 8 (IQR 2-10.5; *W*=244, *P*=.11, *r*=5.083, 95% CI –5 to 1). The Mann-Whitney *U* test showed no significant difference between the groups at baseline (*U*=1191, *P*=.51, *r*=–0.068, 95% CI –2 to 4). Postintervention comparisons approached significance, favoring the intervention group (*U*=869, *P*=.08, *r*=–0.183, 95% CI –6 to 0).

Depression scores, measured using the DASS-21, did not significantly change in the intervention group, with the median remaining at 4 (IQR 2-9.5) for preintervention and 4 (IQR 2-8) for postintervention (*W*=352.5, *P*=.60, *r*=7.663, 95% CI –4 to 4). Although the control group showed an increase in depression scores from a median of 4 (IQR 2-10.5) to 7 (IQR 2-12), this change was not statistically significant (*W*=238, *P*=.09, *r*=4.958, 95% CI –6 to 0). No significant difference between the groups was observed at baseline (*U*=1068.5, *P*=.79, *r*=–0.028, 95% CI –3 to 3), and postintervention group comparisons did not reach statistical significance (*U*=903.5, *P*=.13, *r*=–0.156, 95% CI –6 to 1).

Stress scores, measured using the DASS-21, remained stable in the intervention group, with a median score of 8 (IQR 4-13.5) for preintervention and 8 (IQR 2-13.5) for postintervention time points (*W*=321.5, *P*=.48, *r*=6.989, 95% CI –4 to 5). The control group also showed no significant change in stress scores, with medians of 10 (IQR 4-18) for preintervention and postintervention (IQR 4-16) time points (*W*=387.5, *P*=.98, *r*=8.073, 95% CI –5 to 6). The Mann-Whitney *U* test revealed no significant differences between the groups at baseline (*U*=951.5, *P*=.25, *r*=–0.119, 95% CI –8 to 2), nor postintervention time points (*U*=911.5, *P*=.15, *r*=–0.150, 95% CI –9 to 2).

The intervention group showed no significant changes in EPDS scores, with a median score of 6 (IQR 4-9) for preintervention and 6 (IQR 2-9.75) for postintervention (*W*=272.5, *P*=.16, *r*=5.924, 95% CI –3.5 to 3.0). In the control group, EPDS scores increased slightly from a median of 6 (IQR 2-10.5) to 8 (IQR 3.75-11.25), but this change was not significant (*W*=331.5, *P*=.29, *r*=6.906, 95% CI –5.0 to 2.5). Baseline comparisons showed no significant differences between the groups (*U*=1094.5, *P*=.95, *r*=–0.007, 95% CI –5 to 3), and postintervention group differences were also nonsignificant (*U*=888, *P*=.11, *r*=–0.169, 95% CI –5.0 to 1.5).

**Figure 2:**
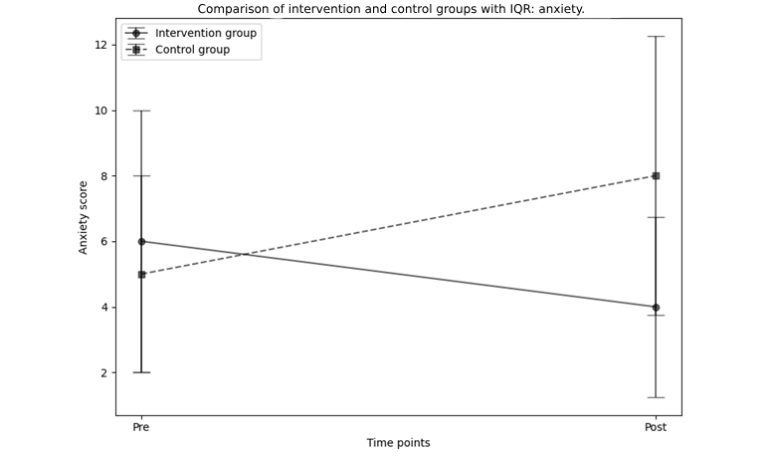
Comparison of median anxiety scores with IQR at pre- and postintervention between intervention and control groups using nonparametric analysis.

The primary outcomes results are summarized in Table 2 and depicted in Figure 2.

#### Secondary Outcomes: Mental Health Well-Being, Maternal-Fetal Attachment, and Mindfulness

The secondary outcomes including changes in well-being, maternal-fetal attachment, and mindfulness are summarized in Table 3. Emotional well-being significantly improved in the intervention group, with the median score increasing from 9 (IQR 8-12) to 11.5 (IQR 8-12) as reflected in the Wilcoxon Signed-Rank test (*W*=154, *P*=.02, effect size *r*=3.348, 95% CI –4.0 to 0.5). No significant changes were observed in the control group over the same period (*W*=287.5, *P*=.47, effect size *r*=5.99, 95% CI –1.5 to 2.5). The Mann-Whitney *U* test postintervention approached significance (*U*=1348, *P*=.07, effect size *r*=–0.19, 95% CI –0.5 to 4.0). According to the FDR correction, the within-group improvement in emotional well-being in the intervention group remained significant, while the between-group difference was not significant.

**Table 3:** Comparison of well-being, maternal-fetal attachment, and mindfulness scores at pre- and postintervention time points between the intervention and control groups using nonparametric analysis.

Scale, variable, and group				Pretest					Posttest					*P* value^a^			*P* value^b^
				Mean (SD)		Median (IQR)		Mean (SD)			Median (IQR)						
**MHC-sf^c^ score**																	
	**Total**																
		IG^d^	35.2 (14.9)		35 (24-48)		38.9 (15.2)			43 (26-49.8)		.07			.06		
		CG^e^	34.8 (16.1)		31.5 (20.8-47.5)		32.3 (16.3)			31.5 (20.8-46)		.34			—^f^		
	**Emotional**																
		IG	9.5 (2.9)		9 (8-12)		10.5 (3.0)			11.5 (8-12)		.02^g^			.07		
		CG	9.4 (3.3)		10 (7-12)		9.0 (3.6)			9 (6-12)		.47			—		
	**Social**																
		IG	9.8 (6.1)		10.5 (5-14)		11.4 (5.9)			11 (8.2-16)		.03			.08		
		CG	10.0 (6.3)		9 (4.8-14.2)		9.4 (5.8)			9.5 (5-14)		.64			—		
	**Psychological**																
		IG	15.9 (6.8)		15.5 (11-21)		17.0 (7.0)			19 (11.2-23)		.34			.06		
		CG	15.5 (7.6)		14.5 (9-21.2)		13.9 (7.6)			13 (7-21)		.19			—		
**MFAS score^h^**																	
	**Total**																
		IG	72.1 (11.2)		72.5 (65-80)		74.5 (11.4)			75.5 (68-83.8)		.15			.18		
		CG	71.1 (11.6)		71.5 (62.8-79.2)		70.9 (12.9)			73 (60.5-82)		.93			—		
	**Differentiation self from fetus**																
		IG	9.0 (1.7)		9 (8-10)		9.8 (1.7)			10 (9-11)		.006^g^			.04		
		CG	9 (2.0)		9 (8-11)		8.9 (2.1)			9 (7-10.2)		.81			—		
	**Interaction with fetus**																
		IG	12.7 (2.4)		13 (11-15)		12.3 (2.4)			13 (10.2-14)		.16			.41		
		CG	12.8 (2.5)		14 (11-15)		12.7 (2.5)			13 (11-15)		.57			—		
	**Attributing characteristics to fetus**																
		IG	20.0 (2.8)		21 (18-22)		21.4 (2.7)			22 (20.2-24)		.003^g^			.002^g^		
		CG	19.4 (3.6)		20 (17-22)		19.5 (3.2)			20 (17.8-22)		.79			—		
	**Role taking**																
		IG	13.1 (2.5)		13 (11-15)		13.4 (2.2)			14 (12-15)		.15			.26		
		CG	13.0 (2.6)		13.5 (11.8-15)		12.7 (2.8)			13 (10.8-16)		.41			—		
	**Giving of self**																
		IG	17.3 (4.2)		17 (14-20.8)		17.6 (4.3)			18 (15-21)		.58			.46		
		CG	16.9 (3.3)		17 (15-19)		17.1 (4.1)			16.5 (14-21)		.62			—		
**CAMS-r^i^ score**																	
	**Total**																
		IG	25.3 (5.9)		25 (21.2-29.8)		26.7 (7.0)			26.5 (21.2-31.8)		.25			.08		
		CG	24.7 (5.4)		26 (21.8-27)		24.2 (5.2)			24 (21-27.2)		.59			—		
	**Awareness**																
		IG	9.9 (2.8)		10 (8-12)		11.2 (3.2)			11 (9-13.8)		.008^g^			.006^g^		
		CG	9.4 (2.1)		9.5 (8.8-11)		9.4 (2.2)			10 (8-11)		.97			—		
	**Acceptance**																
		IG	4.9 (1.5)		5 (4-6)		5.2 (1.5)			5 (4-6)		.23			.21		
		CG	4.8 (1.4)		5 (4-6)		4.8 (1.6)			4.5 (4-6)		.93			—		
	**Attention**																
		IG	10.4 (2.6)		10 (9-12)		10.3 (2.6)			10 (8-12)		.42			.63		
		CG	10.5 (2.6)		10 (9-12.2)		10.0 (2.3)			10 (8-12)		.09			—		

^a^*P* value for Wilcoxon Signed-Rank test for within-group comparisons (pre- to posttest).

^b^*P* value for Mann-Whitney *U* test for between-group comparisons (posttest).

^c^MHC-sf: Mental Health Continuum-Short Form.

^d^IG: intervention group.

^e^CG: control group.

^f^Not applicable.

^g^Significant changes after false discovery rate correction.

^h^MFAS: Maternal-Fetal Attachment scale.

^i^CAMS-r: Cognitive and Affective Mindfulness Scale-revised.

Differentiation self from fetus dimension of maternal-fetal attachment significantly improved in the intervention group, with median scores increasing from 9 (IQR 8-10) to 10 (IQR 9-11), as reflected in the Wilcoxon Signed-Rank test (*W*=129.5, *P*=.006, effect size *r*=2.815, 95% CI –1.5 to 0.0). No significant changes were found in the control group (*W*=206.5, *P*=.81, effect size *r*=4.302, 95% CI –0.5 to 0.0). Postintervention comparisons revealed a significant difference between groups, favoring the intervention group (*U*=1379, *P*=.04, effect size *r*=–0.215, 95% CI 0.0-1.5). After FDR correction, the within-group improvement in the intervention group remained significant, while the between-group difference was not significant.

The attributing characteristics to fetus dimension of maternal-fetal attachment significantly improved within the intervention group, with median scores rising from 21 (IQR 18-22) to 22 (IQR 20.25-24), as indicated by the Wilcoxon Signed-Rank test (*W*=107, *P*=.003, effect size *r*=2.326, 95% CI –3 to 0). No significant changes were observed in the control group (*W*=316, *P*=.79, effect size *r*=6.583, 95% CI –2.0 to 2.5). The Mann-Whitney *U* test revealed a significant postintervention difference between the groups, favoring the intervention group (*U*=1506.5, *P*=.002, effect size *r*=–0.314, 95% CI 0.5-4.0). After FDR correction, both the within-group improvement in the intervention group and the between-group difference remained significant.

Awareness from CAMS-r showed a significant increase in the intervention group from pre- to postintervention time points. The median score rose from 10 (IQR 8-12) to 11 (IQR 9-13.75), as indicated by the Wilcoxon Signed-Rank test (*W*=200, *P*=.008, effect size *r*=4.348, 95% CI –3.0 to 0.5). In contrast, the control group did not exhibit significant changes in awareness over the same period (*W*=406.5, *P*=.97, effect size *r*=8.469, 95% CI –1 to 1). Postintervention comparisons between the groups revealed a significant difference favoring the intervention group (*U*=1467.5, *P*=.006, effect size *r*=–0.284, 95% CI 0-3). The FDR correction confirmed that both the within-group improvement and the between-group difference were significant.

Subscales within the secondary outcomes, such as (1) social and psychological well-being (MHC-sf); (2) interaction with the fetus, giving of self, and maternal role-taking (MFAS); and (3) attention and acceptance in mindfulness (CAMS-r), did not show significant changes postintervention, as indicated by the FDR-adjusted analyses.

The comprehensive effects of both primary and secondary outcomes, including statistical significance and CIs, are detailed in Table 4.

**Table 4:** Effect sizes and 95% CIs for significant primary and secondary outcomes, comparing intervention and control groups.

Variable and group		Within comparison			Between comparison	
		Effect Size *r* (95% CI)	*P* value	Effect size *r* (95% CI)		*P* value
**Anxiety**						
	IG^a^	6.413 (–2.0 to 5.0)	.03	–0.183 (–6.0 to 0.0)		.08
	CG^b^	5.083 (–5.0 to 1.0)	.10	—^c^		—
**Emotional well-being**						
	IG	3.348 (–4.0 to 0.5)	.01	–0.19 (–0.5 to 4.0)		.07
	CG	5.99 (–1.5 to 2.5)	.47	—		—
**Differentiation self from fetus**						
	IG	2.815 (–1.5 to 0.0)	.006	–0.215 (0.0 to 1.5)		.04
	CG	4.302 (–0.5 to 0.0)	.81	—		—
**Attributing characteristics to fetus**						
	IG	2.326 (–3.0 to 0.0)	.003	–0.314 (0.5 to 4.0)		.002
	CG	6.583 (–2.0 to 2.5)	.79	—		—
**Awareness**						
	IG	4.348 (–3.0 to 0.5)	.008	–0.284 (0.0 to 3.0)		.006
	CG	8.469 (–1.0 to 1.0)	.96	—		—

^a^IG: intervention group.

^b^CG: control group.

^c^Not applicable.

#### Usability

Of the 133 participants, 80 responded to the USE questionnaire. Around 88% (70/80) of participants strongly agreed to the usability of the app. The total mean score was 5.7 (SD 0.62). Around 75% (60/80) of participants agreed regarding the app’s usefulness. The mean score of usefulness was 5.4 (SD 0.79); 98% (78/80) of participants agreed that the app was easy to learn from. The mean score of ease of learning was 6.4 (SD 0.67); 91% (73/80) of participants agreed that the app was easy to use. The mean ease of use score was 5.9 (SD 0.68). In addition, 81% (65/80) of participants agreed with the satisfaction of the app. The mean score of the satisfaction was 5.6 (SD 0.79).

## Discussion

### Principal Findings

This study supports the effectiveness of the mobile-based mindfulness intervention developed for pregnant women in reducing perinatal anxiety. Furthermore, the intervention enhanced emotional well-being, maternal-fetal attachment, and mindful awareness during pregnancy. While previous studies have demonstrated the benefits of MBIs for perinatal mental health [25,31], our study is among the first to evaluate a fully self-guided, short-term (4-week) mobile intervention without the need for in-person guidance. This distinguishes our work as a widely applicable and accessible approach, particularly suited for populations with limited access to traditional mental health services. The findings provide evidence for both the effectiveness and usability of the mobile app as a tool for promoting mental health during pregnancy.

Perinatal anxiety was significantly reduced in pregnant women who used the self-administered mobile mindfulness program compared with the control group. Anxiety during pregnancy has been linked to various adverse outcomes, including mental health issues (such as postnatal depression) and perinatal complications (such as preterm birth). Our findings suggest that this intervention can help prevent and manage anxiety before it escalates to a clinical level. Even when anxiety is not at a clinical threshold, it can still have a significant impact, and interventions like ours can play an important role in addressing and controlling it early on. In a randomized controlled trial by Sun et al [33], there was a significant reduction in anxiety in pregnant women at risk of perinatal depression using an 8-week smartphone-based mindfulness training. Compared with these high-anxiety participants, those in this study were a general pregnant population with lower baseline anxiety levels. Our findings suggest that the mobile mindfulness intervention can effectively reduce anxiety even in a general pregnancy population.

Our 4-week intervention was designed to be more feasible and accessible, addressing the need for shorter, yet effective, interventions. This study stands out due to its shorter duration compared with similar interventions, such as the 8-week program used in previous studies [18,33]. A full 8-week MBCT program may provide a more comprehensive intervention for those willing to participate. In reality, however, committing to a long-term regular in-person treatment during pregnancy may be very challenging with demands on the time and cost of the pregnant mothers, along with other barriers to treatment including the scarcity of perinatal treatment resources and stigma of going to psychiatric clinics. In previous research, the mean number of completed training weeks of the 8-week intervention was 3 [33]. Mobile-based or internet-based forms of treatment delivery may help overcome these limitations and promote access to timely interventions. Our study demonstrates that an MBCT-modified, short-term, self-administered mobile mindfulness intervention may benefit mental health during the perinatal period.

An analysis of the secondary outcomes from the mobile mindfulness intervention program revealed significant improvements in emotional well-being, a subscale of the well-being questionnaire (MHC-sf), among pregnant women following the 4-week intervention. Previous research has shown that poor maternal emotional well-being is associated with childbirth complications, such as low birth weight and fetal developmental issues, as well as long-term cognitive and behavioral problems in the child [56]. Enhanced emotional well-being has also been linked to better physical and mental health outcomes for pregnant women, improved labor outcomes, better health outcomes for offspring, and stronger maternal attachment [27,56]. Our findings suggest that mobile mindfulness training has the potential to promote emotional well-being during pregnancy and could serve as an accessible and effective tool for supporting mental health during this challenging period. In addition, the significant improvements in emotional well-being observed in the intervention group reinforce the effectiveness of MBIs in enhancing mental well-being, even in populations not at high risk for mental health disorders [56].

Another notable finding from our study was the positive effect of the mobile program on maternal-fetal attachment. Attachment, or the relationship between the mother and her infant, begins before birth (during pregnancy) as a result of dynamic psychological and physiological events. During pregnancy, women have intellectual, physical, and kinesthetic awareness of the fetus. Gradually, they become aware of the fetus as a separate individual. Women notice and interpret different movements of the fetus, elaborating personal relationships. Using the MFAS, domains of differentiation from the fetus and attribution of characteristics to the fetus were significantly increased in the intervention group. Differentiation of the self from the fetus indicates that the woman recognizes the fetus as an individual separate from her body [57]. The ability to attribute characteristics, such as personality, emotions, and physical space in the uterus, to the fetus is part of the dynamic relationship between mother and fetus [48,57]. Increased scores on these characteristics mean that mothers have a high level of attachment with their fetuses, by forming an identity as a mother and recognizing their developing fetuses; in turn, they may be more prepared and invested in their pregnancy [49]. Being in a mindful state means being aware of both the self and others, as well as one’s inner and outer self. Mobile-based mindfulness training may lead to an increased awareness of the fetus as an individual, leading to qualitative changes in the interaction with the fetus. Improved maternal-fetal attachment has long-term benefits, such as a reduction in postpartum depression [57]. This study shows that a self-administered mindfulness program may promote maternal-fetal attachment, even in a relatively short duration of 4 weeks. Whether these effects may have a lasting, long-term benefit should be evaluated in the future.

Increased mindful awareness was shown in the intervention group, and supporting the newly developed 4-week mobile mindfulness program may cultivate or increase skills of mindfulness. Mindful awareness is nonjudgmental and nonreactive attention to experiences occurring at the present moment, including cognition, emotion, and bodily sensations, as well as surrounding environmental stimuli [58]. When one is mindfully aware, adaptation to stressful situations increases by enhancing attention to the negative emotional states [59]. Promotion of actual skills may have more persisting benefits, as these skills can be used in everyday life. Our findings suggest that these skills can be fostered through mobile-based, self-administration, even within a relatively short period of 4 weeks. Maintaining awareness, without judgment or reactivity, toward emotions about the dramatic changes that come with childbirth and related beliefs or automatic thoughts, may help in emotion regulation and behavioral adaptation. In a previous study that investigated the effect of the in-person MBCT program for 9 weeks increased natural childbirths for pregnant women with high fears of childbirth, increased mindful awareness was the most powerful mechanism of change to better adapt to the challenges of childbirth [60]. The present mobile intervention may be an accessible tool to effectively cultivate mindful awareness to increase tolerance to negative emotional states and cope with them better.

The mindfulness program scored high on usefulness, ease of use, ease of learning, ease to use, and overall satisfaction. Previously, it was noted that the traditional mindfulness intervention was challenging to access for women who already had children because of time limitations and existing family responsibilities, which led to a high loss to follow-up [60]. While mobile apps may be a preferable mode of delivery for mental health interventions to mitigate these barriers, the benefits of the intervention may be limited if it is difficult for users to administer. Current findings suggest that the mobile program is both feasible and acceptable. Furthermore, the responses from the usability study provided useful information for future program modifications. The mobile app used in our study automatically provides push notification services to serve the purpose of the mindfulness intervention program (eg, a reminder to use the app at least twice a week). This feature helped improve adherence for some participants. Features like these within a mobile app have the potential to enhance adherence and effectiveness, while a user-friendly design can facilitate the broad and efficient implementation of the app to provide motivation for continued use [61].

### Limitations

Several limitations should be acknowledged. First, we only found results from the immediate effects of postintervention because our study design did not include a long-term follow-up. Future research should explore the long-term effects, potentially including postpartum outcomes for both the mother and infant, to better inform perinatal care. Given the relatively short study period (4 weeks), it is important to determine whether the observed benefits are long-term, or if additional or booster sessions might be necessary to maintain or enhance these benefits. Second, our sample consisted of a general pregnancy population, and thus, the results of the current study do not represent effectiveness in a clinical sample such as that of a depressive disorder group [25]. No significant effect was observed on depression scores in this study, which may be attributed to the low scores that represent no clinical depression at baseline. It will be helpful to investigate whether the intervention can reduce clinical levels of anxiety or depression in the future. Finally, the effect sizes of depression, anxiety, and stress in this study were small, potentially due to the limited sample size. This also suggests that outcomes such as depression and stress may have shown no significant effect due to insufficient power. Future studies should aim to recruit a larger sample size to ensure adequate power to detect significant effects.

### Conclusions

Mindfulness-based mobile interventions offer an effective tool for alleviating prenatal anxiety and improving emotional well-being as well as maternal-fetal attachment. This intervention may serve as a cost-effective and highly accessible method of intervention to overcome the current limitations of perinatal mental health management. Further research is needed to investigate whether the program provides lasting benefits for both mother and infant.

## Data Availability

The datasets generated and/or analyzed during this study are not publicly available because of restrictions containing information that could compromise the privacy of study participants but are available from the corresponding author upon reasonable request.

## Appendix

Multimedia Appendix 1 CONSORT-eHEALTH checklist (V 1.6.2).

Multimedia Appendix 2 Overview of the mindfulness-based intervention AvecMom mobile app content.

Multimedia Appendix 3 Screenshots of AvecMom mindfulness mobile app.

## Abbreviations

CAMS-r: Cognitive and Affective Mindfulness Scale-revised
CONSORT-EHEALTH: Consolidated Standards of Reporting Trials of Electronic and Mobile Health Applications and Online Telehealth
DASS-21: 21-item Depression, Anxiety and Stress Scale
EPDS: Edinburgh Postnatal Depression Scale
FDR: false discovery rate
MBCT: mindfulness-based cognitive therapy
MBI: mindfulness-based intervention
MFAS: Maternal-Fetal Attachment scale
MHC-sf: Mental Health Continuum-Short Form
USE: Usefulness, Satisfaction, and Ease of Use
