# Supplementary material for: Development and Evaluation of a Mindfulness-Based Mobile Intervention for Perinatal Mental Health: Randomized Controlled Trial
Source: J Med Internet Res. 2025 Jan 17;27:e56601. doi: 10.2196/56601 (PMC11786135; doi:10.2196/56601)
Supplement: Multimedia Appendix 1 [file jmir_v27i1e56601_app1.pdf]

CONSORT-EHEALTH Checklist V1.6.2 Report	Manuscript Number	56601
**(based on CONSORT-EHEALTH V1.6), available at [http://tinyurl.com/consort-ehealth-v1-6].**		
**Date completed**		
11/27/2024 0:51:38		
**by**		
Sehwan		
TITLE		
**1a-i) Identify the mode of delivery in the title**		
**1a-ii) Non-web-based components or important co-interventions in title**		
"Development and Evaluation of a Mindfulness-Based Mobile Intervention for Perinatal Mental Health: A Randomized Controlled Trial"		
**1a-iii) Primary condition or target group in the title**		
ABSTRACT		
**1b-i) Key features/functionalities/components of the intervention and comparator in the METHODS section of the ABSTRACT**		
**1b-ii) Level of human involvement in the METHODS section of the ABSTRACT**		
"The intervention group participated in a self-administered 4-week smartphone-based mindfulness program."<br>"Pregnant women were recruited and randomized to an intervention or a wait-list control group."<br>"Anxiety, depression, and stress were assessed as primary outcomes at baseline and post-intervention. Secondary outcomes were mental health well-being, maternal-fetal attachment, and skills of mindfulness. The usability of the mobile intervention was also evaluated."		
**1b-iii) Open vs. closed, web-based (self-assessment) vs. face-to-face assessments in the METHODS section of the ABSTRACT**		
"The intervention group participated in a self-administered 4-week smartphone-based mindfulness program."		
**1b-iv) RESULTS section in abstract must contain use data**		
"Pregnant women were recruited and randomized to an intervention or a wait-list control group. The intervention group participated in a self-administered 4-week smartphone-based mindfulness program. Anxiety, depression, and stress were assessed as primary outcomes at baseline and post-intervention."		
**1b-v) CONCLUSIONS/DISCUSSION in abstract for negative trials**		
"A total of 133 pregnant women were randomly assigned to the intervention (n=66) or the control group (n=67). The overall dropout rate was 30% (39/133)."		
INTRODUCTION		
**2a-i) Problem and the type of system/solution**		
**2a-ii) Scientific background, rationale: What is known about the (type of) system**		
"Perinatal women are reported to be at a higher risk of developing newly-onset mental health problems or experiencing a relapse of existing ones compared to the general population."<br>"Despite the significant impact of perinatal mental health issues, many women face barriers that prevent them from accessing necessary mental health care. In obstetric clinics, routine mental health evaluations for anxiety, depression, and stress are often neglected or overlooked, and it is uncommon for pregnant women to seek mental health care directly from psychiatric services."<br>"Given these barriers, there is a clear need for effective and more accessible non-pharmacological interventions for women during this critical period, including digital and mobile-based programs, that can overcome obstacles like stigma and limited resources."		
METHODS		
**3a) CONSORT: Description of trial design (such as parallel, factorial) including allocation ratio**		
Checklist submission was hindered by the character limit, and identifying the problematic sections was not straightforward, resulting in numerous edits and added frustration.		
**3b) CONSORT: Important changes to methods after trial commencement (such as eligibility criteria), with reasons**		
"In this randomized controlled trial (RCT), we evaluated the effectiveness of our newly developed, 4-week mindfulness-based mobile application in reducing perinatal anxiety, depression, and stress among a general population of pregnant women. We also investigated mental health well-being, maternal-fetal attachment, and mindfulness skills as secondary outcomes, and assessed the usability of the intervention to determine feasibility for broader application."		
**3b-i) Bug fixes, Downtimes, Content Changes**		
**4a) CONSORT: Eligibility criteria for participants**		
"This study was a single-center, randomized wait-list controlled trial with assessments at baseline and post-intervention (trial registration: KCT0007166). Participants were randomized using a list created in the R (4.0.2 version) program, which was set to randomize in a 1:1 ratio."		
**4a-i) Computer / Internet literacy**		
**4a-ii) Open vs. closed, web-based vs. face-to-face assessments:**		
"The inclusion criteria were as follows: (1) at least 18 years of age, (2) between 1 and 32 weeks gestation, (3) having a smartphone, (4) being able to use the app on a smartphone for the study, (5) being able to read and understand Korean, (6) willing to be randomized, and (7) willing to provide informed consent."		
**4a-iii) Information giving during recruitment**		
"Participants were recruited both from online advertisement and on-site at the Obstetrics and Gynecology Department of the CHA Bundang Medical Center in Seongnam, South Korea, from March to November 2020."		
**4b) CONSORT: Settings and locations where the data were collected**		
There were no changes to the methods, including eligibility criteria, after the trial commenced.		
**4b-i) Report if outcomes were (self-)assessed through online questionnaires**		
**4b-ii) Report how institutional affiliations are displayed**		
"If participants could not visit to the obstetric department or complete the questionnaire at the site, they received a Google survey link of the questionnaire via email or smartphone."		
**5) CONSORT: Describe the interventions for each group with sufficient details to allow replication, including how and when they were actually administered**		
**5-i) Mention names, credential, affiliations of the developers, sponsors, and owners**		
**5-ii) Describe the history/development process**		
"The mindfulness-based mobile intervention was developed by an interdisciplinary research team. The development was led by a professor of psychiatry who has over five years of mindfulness experience as well as experience with leading the development of several preventive interventions for mental health-related at-risk populations. The team also consisted of a psychiatrist with expertise in mobile applications, obstetricians, psychologists, a research professor of user interface and user experience design, and research assistants with mindfulness experience."		
**5-iii) Revisions and updating**		
"The mindfulness-based mobile intervention was developed by an interdisciplinary research team. The development was led by a professor of psychiatry who has over five years of mindfulness experience as well as experience with leading the development of several preventive interventions for mental health-related at-risk populations."<br>"The development process of the intervention began with a comprehensive review of previous mindfulness-based interventions to identify essential components relevant to perinatal mental health [37]. Following this, we focused on component selection, choosing mindfulness practices that address perinatal stress, anxiety, and depression, with content tailored to meet the unique physical, social, and psychological needs of pregnant women."		
**5-iv) Quality assurance methods**		

Minor bug fixes were implemented during the trial to address server-side issues. These fixes were technical adjustments that did not affect participants' access, usage, or the intervention content.		
**5-v) Ensure replicability by publishing the source code, and/or providing screenshots/screen-capture video, and/or providing flowcharts of the algorithms used**		
"Finally, the program underwent expert review by a panel of experienced advisors in mindfulness and perinatal mental health, whose insights helped refine the content and structure. The recorded sessions were guided by an experienced mindfulness trainer who was also pregnant at the time, strengthening the intervention's relevance and the empathetic element to the intervention. Participants were reminded that this app is not equivalent to psychotherapy and were recommended to seek professional help when necessary."		
**5-vi) Digital preservation**		
"Multimedia Appendix 2. Screenshots of AvecMom mindfulness mobile app."		
**5-vii) Access**		
**5-viii) Mode of delivery, features/functionalities/components of the intervention and comparator, and the theoretical framework**		
"Participants assigned to the intervention group were instructed to download a mobile-based mindfulness intervention application named AvecMom and were instructed to use it for four weeks. They received printed guides on how to use the mobile app and direct download links for both Android and iPhone devices."		
**5-ix) Describe use parameters**		
"The mindfulness-based mobile intervention was developed by an interdisciplinary research team. The development was led by a professor of psychiatry who has over five years of mindfulness experience as well as experience with leading the development of several preventive interventions for mental health-related at-risk populations."<br>"the program underwent expert review by a panel of experienced advisors in mindfulness and perinatal mental health, whose insights helped refine the content and structure. The recorded sessions were guided by an experienced mindfulness trainer who was also pregnant at the time, strengthening the intervention's relevance and the empathetic element to the intervention. Participants were reminded that this app is not equivalent to psychotherapy and were recommended to seek professional help when necessary."<br>"The intervention program consisted of four sections: breathing mindfulness meditation, body scanning, emotional awareness, and self-kindness mindfulness (Supplementary material 1)."<br>"Each session had a duration of about 20 minutes. Participants were instructed to practice each session at least twice, ensuring they engaged with the content multiple times to reinforce their learning and practice."		
**5-x) Clarify the level of human involvement**		
"Each session had a duration of about 20 minutes. Participants were instructed to practice each session at least twice, ensuring they engaged with the content multiple times to reinforce their learning and practice. This structured approach aimed to enhance usability and effectiveness, providing a comprehensive yet manageable intervention for pregnant women. Sessions took into consideration the prenatal-specific psychological, physical, and emotional changes specific to pregnancy."		
**5-xi) Report any prompts/reminders used**		
"Participants assigned to the intervention group were instructed to download a mobile-based mindfulness intervention application named AvecMom and were instructed to use it for four weeks. They received printed guides on how to use the mobile app and direct download links for both Android and iPhone devices. Participants were instructed to practice each practice session at least three times a week for the four weeks of participating in the program."		
**5-xii) Describe any co-interventions (incl. training/support)**		
The app included an optional in-app reminder feature that allowed users to set alerts to engage with the intervention. This feature was participant-driven, and its use was not mandatory or monitored during the trial. The timing and frequency of reminders were customizable by the users.		
**6a) CONSORT: Completely defined pre-specified primary and secondary outcome measures, including how and when they were assessed**		
"The inclusion criteria were as follows: (1) at least 18 years of age, (2) between 1 and 32 weeks gestation, (3) having a smartphone, (4) being able to use the app on a smartphone for the study, (5) being able to read and understand Korean, (6) willing to be randomized, and (7) willing to provide informed consent. Exclusion criteria were: (1) over 32 weeks of gestation, (2) expected to give birth within 4 weeks (during the intervention period). The study did not exclude participants based on medical history (e.g., obstetric complications or mental illness) unless these conditions reached a clinically significant level that would affect participation or safety of the participant."		
**6a-i) Online questionnaires: describe if they were validated for online use and apply CHERRIES items to describe how the questionnaires were designed/deployed**		
**6a-ii) Describe whether and how "use" (including intensity of use/dosage) was defined/measured/monitored**		
"If participants could not visit to the obstetric department or complete the questionnaire at the site, they received a Google survey link of the questionnaire via email or smartphone."		
**6a-iii) Describe whether, how, and when qualitative feedback from participants was obtained**		
**6b) CONSORT: Any changes to trial outcomes after the trial commenced, with reasons**		
"Participants were recruited both from online advertisement and on-site at the Obstetrics and Gynecology Department of the CHA Bundang Medical Center in Seongnam, South Korea, from March to November 2020."		
**7a) CONSORT: How sample size was determined**		
**7a-i) Describe whether and how expected attrition was taken into account when calculating the sample size**		
**7b) CONSORT: When applicable, explanation of any interim analyses and stopping guidelines**		
"Depression, anxiety, and stress were assessed with the Depression, Anxiety and Stress Scale, 21-question version (DASS-21) [39, 40, 41]. This scale consists of 21 items used to assess depression (seven items), anxiety (seven items), and stress (seven items). The response items were scored on a four-point Likert scale ranging from 0 to 3. Higher scores for each factor indicate a higher intensity for each symptom. A depression score of 9 or higher, an anxiety score of 7 or higher, and a stress score of 14 or higher indicate moderate symptoms for each factor [40]. The internal reliability for DASS-21 was found to be high (α= 0.94, at baseline)."<br>Secondary outcomes were assessed at baseline and post-intervention and included: MHC-sf, MFAS, CAMS-r.		
**8a) CONSORT: Method used to generate the random allocation sequence**		
There were no changes to the trial outcomes after the trial commenced.		
**8b) CONSORT: Type of randomisation; details of any restriction (such as blocking and block size)**		
There were no interim analyses or stopping guidelines specified or implemented during this trial. The study followed the pre-specified protocol until its conclusion.		
**9) CONSORT: Mechanism used to implement the random allocation sequence (such as sequentially numbered containers), describing any steps taken to conceal the sequence until interventions were assigned**		
"Participants were randomized using a list created in the R (4.0.2 version) program, which was set to randomize in a 1:1 ratio. To ensure the integrity of the data analysis and eliminate potential bias, the data analysts remained blinded to the group allocation of each participant. The randomization was performed by a designated member of the research team who was not involved in the data analysis. The randomization sheet was only accessible to the person responsible for assigning participants to their respective groups."		
**10) CONSORT: Who generated the random allocation sequence, who enrolled participants, and who assigned participants to interventions**		
"Participants were randomized using a list created in the R (4.0.2 version) program, which was set to randomize in a 1:1 ratio."		
**11a) CONSORT: Blinding - If done, who was blinded after assignment to interventions (for example, participants, care providers, those assessing outcomes) and how**		
**11a-i) Specify who was blinded, and who wasn't**		
**11a-ii) Discuss e.g., whether participants knew which intervention was the "intervention of interest" and which one was the "comparator"**		
"To ensure the integrity of the data analysis and eliminate potential bias, the data analysts remained blinded to the group allocation of each participant. The randomization was performed by a designated member of the research team who was not involved in the data analysis. The randomization sheet was only accessible to the person responsible for assigning participants to their respective groups."		
**11b) CONSORT: If relevant, description of the similarity of interventions**		
"The randomization sheet was only accessible to the person responsible for assigning participants to their respective groups. A designated member of the research team allocated the participants to the intervention or control group using the randomized list. Those randomly assigned to the wait-list control group received the intervention after four weeks."		

**12a) CONSORT: Statistical methods used to compare groups for primary and secondary outcomes**		
"Figure 1. Participant enrollment flow chart."		
**12a-i) Imputation techniques to deal with attrition / missing values**		
**12b) CONSORT: Methods for additional analyses, such as subgroup analyses and adjusted analyses**		
This item is not applicable, as the study did not involve a placebo or sham intervention.		
RESULTS		
**13a) CONSORT: For each group, the numbers of participants who were randomly assigned, received intended treatment, and were analysed for the primary outcome**		
**13b) CONSORT: For each group, losses and exclusions after randomisation, together with reasons**		
The study did not include subgroup or adjusted analyses. All statistical analyses were conducted as specified in the trial protocol, focusing on primary and secondary outcomes using nonparametric methods		
**13b-i) Attrition diagram**		
**14a) CONSORT: Dates defining the periods of recruitment and follow-up**		
"A total of 143 perinatal women were recruited as eligible for the study. Of the 143 participants, 2% (3/143) did not meet the inclusion criteria, and 5% (7/143) did not complete the baseline assessment."		
**14a-i) Indicate if critical "secular events" fell into the study period**		
**14b) CONSORT: Why the trial ended or was stopped (early)**		
"A total of 143 perinatal women were recruited as eligible for the study. Of the 143 participants, 2% (3/143) did not meet the inclusion criteria, and 5% (7/143) did not complete the baseline assessment. Thus, 133 participants were allocated randomly, of which 66 were allocated to the intervention group and 67 to the wait-list control group. During the follow-ups, 13% (17/133) did not complete the follow-up assessment and 17% (22/133) were not willing to continue participation. The overall completion rate was 71% (94/133)."		
**15) CONSORT: A table showing baseline demographic and clinical characteristics for each group**		
"Participants were recruited both from online advertisement and on-site at the Obstetrics and Gynecology Department of the CHA Bundang Medical Center in Seongnam, South Korea, from March to November 2020."		
**15-i) Report demographics associated with digital divide issues**		
**16a) CONSORT: For each group, number of participants (denominator) included in each analysis and whether the analysis was by original assigned groups**		
**16-i) Report multiple "denominators" and provide definitions**		
**16-ii) Primary analysis should be intent-to-treat**		
"A total of 143 perinatal women were recruited as eligible for the study. Of the 143 participants, 2% (3/143) did not meet the inclusion criteria, and 5% (7/143) did not complete the baseline assessment. Thus, 133 participants were allocated randomly, of which 66 were allocated to the intervention group and 67 to the wait-list control group. During the follow-ups, 13% (17/133) did not complete the follow-up assessment and 17% (22/133) were not willing to continue participation. The overall completion rate was 71% (94/133)."<br>"Figure 1. Participant enrollment flow chart."		
**17a) CONSORT: For each primary and secondary outcome, results for each group, and the estimated effect size and its precision (such as 95% confidence interval)**		
The trial was conducted as planned and was not stopped early.		
**17a-i) Presentation of process outcomes such as metrics of use and intensity of use**		
**17b) CONSORT: For binary outcomes, presentation of both absolute and relative effect sizes is recommended**		
"Table 1. Demographics and clinical characteristics of study participants (n=133)"		
**18) CONSORT: Results of any other analyses performed, including subgroup analyses and adjusted analyses, distinguishing pre-specified from exploratory**		
The manuscript includes comprehensive results for each primary and secondary outcome, with detailed group comparisons, estimated effect sizes, and 95% confidence intervals.		
**18-i) Subgroup analysis of comparing only users**		
**19) CONSORT: All important harms or unintended effects in each group**		
"Anxiety scores, measured using the DASS-21, significantly decreased in the intervention group from pre- to post-intervention, with the median score dropping from 6 (IQR: 2-10) to 4 (IQR: 2-7.5), as shown by the Wilcoxon Signed-Rank test (W=295, p=.030, r=6.413, 95% CI [−2.0, 5.0]). In contrast, no significant changes were observed in the control group, where anxiety scores increased from a median of 5 (IQR: 2-8) to 8 (IQR: 2-10.5) (W=244, p=.103, r=5.083, 95% CI [−5.0, 1.0])."		
**19-i) Include privacy breaches, technical problems**		
**19-ii) Include qualitative feedback from participants or observations from staff/researchers**		
No privacy breaches or significant technical problems occurred during the trial.		
DISCUSSION		
**20) CONSORT: Trial limitations, addressing sources of potential bias, imprecision, multiplicity of analyses**		
**20-i) Typical limitations in ehealth trials**		
**21) CONSORT: Generalisability (external validity, applicability) of the trial findings**		
**21-i) Generalizability to other populations**		
**21-ii) Discuss if there were elements in the RCT that would be different in a routine application setting**		
"our sample consisted of a general pregnancy population, and thus, the results of the current study do not represent effectiveness in a clinical sample such as that of a depressive disorder group [25]."		
**22) CONSORT: Interpretation consistent with results, balancing benefits and harms, and considering other relevant evidence**		
**22-i) Restate study questions and summarize the answers suggested by the data, starting with primary outcomes and process outcomes (use)**		
**22-ii) Highlight unanswered new questions, suggest future research**		
The study aimed to evaluate the effectiveness of a 4-week mobile mindfulness intervention for pregnant women in reducing anxiety, depression, and stress while improving emotional well-being, maternal-fetal attachment, and mindfulness skills. Primary outcomes showed significant reductions in anxiety and improvements in emotional well-being in the intervention group, while depression and stress did not show significant changes.		
Other information		
**23) CONSORT: Registration number and name of trial registry**		
Due to the non-normal distribution of the primary and secondary outcome data, nonparametric methods were applied. The Mann-Whitney U test was employed to compare outcomes between the Intervention and Control groups, while within-group changes from pre- to post-intervention were assessed using the Wilcoxon Signed-Rank test.<br>Subscales within the secondary outcomes, such as social and psychological well-being (MHC-sf), interaction with the fetus, giving of self, and maternal role-taking (MFAS), and attention and acceptance in mindfulness (CAMS-r), did not show significant changes post-intervention, as indicated by the FDR-adjusted analyses.		
**24) CONSORT: Where the full trial protocol can be accessed, if available**		
No harms or unintended effects were reported in either group during the trial period.		
**25) CONSORT: Sources of funding and other support (such as supply of drugs), role of funders**		

"Ethical Consideration<br>This study was approved by the Institutional Review Board, CHA Bundang Medical Center, CHA University College of Medicine (HI18C0911)."		
**X26-i) Comment on ethics committee approval**		
**x26-ii) Outline informed consent procedures**		
"Ethical Consideration<br>This study was approved by the Institutional Review Board, CHA Bundang Medical Center, CHA University College of Medicine (HI18C0911)."		
**X26-iii) Safety and security procedures**		
"All participants provided written informed consent prior to enrollment. Participants were fully informed about the study's purpose, procedures, potential risk and their right to withdraw at any time without consequence. To ensure privacy and confidentiality, all data were de-identified before analysis and securely stored on encrypted servers, accessible only to authorized research staff."		
**X27-i) State the relation of the study team towards the system being evaluated**		
