# Supplementary material for: Development and Evaluation of a Mindfulness-Based Mobile Intervention for Perinatal Mental Health: Randomized Controlled Trial
Source: J Med Internet Res. 2025 Jan 17;27:e56601. doi: 10.2196/56601 (PMC11786135; doi:10.2196/56601)
Supplement: Multimedia Appendix 2 [file jmir_v27i1e56601_app2.docx]

**Multimedia Appendix**

Supplementary material 2. Overview of the mindfulness-based intervention *AvecMom* mobile app content

| Week | Theme | Curriculum | Training |
| --- | --- | --- | --- |
| 1 | Breathing mindfulness meditation | • How to do mindful breathing  • Recognizing negative emotions and uncomfortable sensations during pregnancy | Mindful breathing |
| 2 | Body scan | • Being in the present moment  • Being aware of characteristics body sensations in pregnancy  • Paying attention to sensations throughout the whole body | Body scan |
| 3 | Emotional mindfulness | • Being in the present moment  • Being aware of the degrees of emotions (e.g. joy, satisfaction, comfort)  • Practice accepting current feelings | Mindful meditation |
| 4 | Self-Kindness mindfulness | • Being aware of the current state of the fetus and self  • Having compassion for self and others | Mindful meditation |
