# Supplementary figures and images for: Development and Evaluation of a Mindfulness-Based Mobile Intervention for Perinatal Mental Health: Randomized Controlled Trial
Source: J Med Internet Res. 2025 Jan 17;27:e56601. doi: 10.2196/56601 (PMC11786135; doi:10.2196/56601)

**Multimedia Appendix**

Supplementary material 3. Screenshots of AvecMom mindfulness mobile app


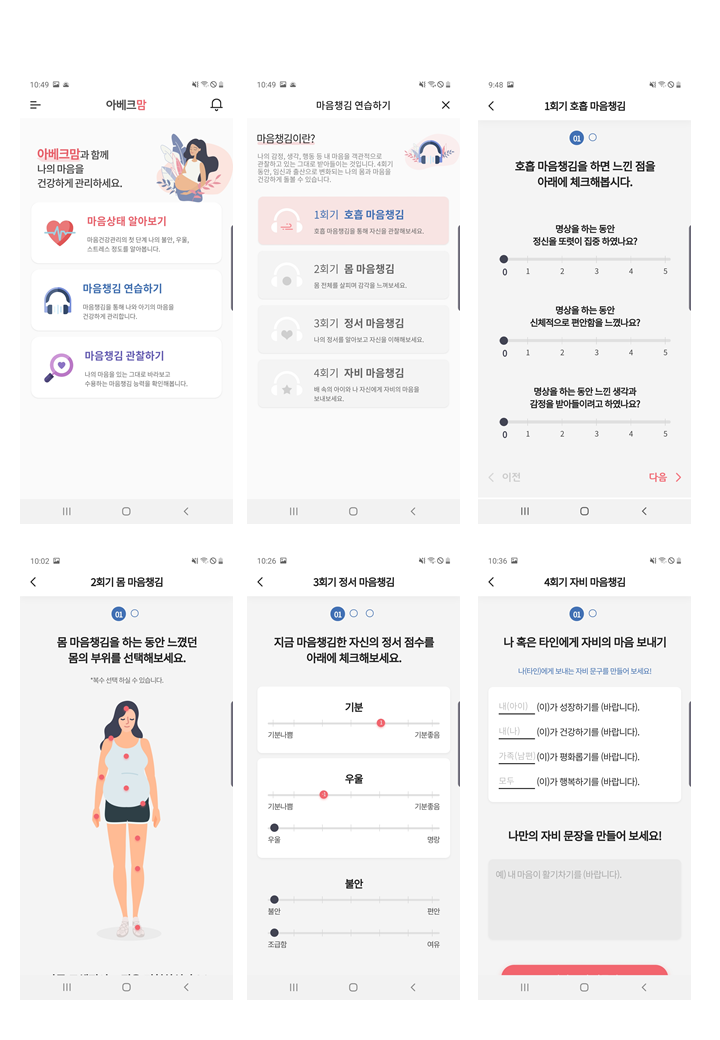

Supplement: Multimedia Appendix 3 [file jmir_v27i1e56601_app3.docx]
